# Active Tablet Coating with Amorphous Solid Dispersion of Ibuprofen–HPMCAS from Organic Solution

**DOI:** 10.3390/pharmaceutics17121514

**Published:** 2025-11-24

**Authors:** Liene Raciborska, Elżbieta Maria Buczkowska, Kirils Kukuls, Līga Pētersone, Valentyn Mohylyuk

**Affiliations:** Leading Research Group, Faculty of Pharmacy, Riga Stradiņš University, 21 Konsula Street, LV-1007 Riga, Latvia

**Keywords:** tablets, active coating, tablet coating, dosing, drug loading, dosage form design, fixed-dose combination

## Abstract

**Background/Objectives:** As a formulation strategy to produce fixed-dose combinations with amorphous solid dispersions of poorly soluble drugs, active coating of tablets is an under-investigated topic. **Methods:** In this study, ibuprofen, with a boiling point of 157 °C, was used as a model drug, and an ibuprofen–HPMCAS coating was loaded on the surface of placebo tablets using a widely used perforated laboratory pan-coater with a single two-component nozzle. Acetone, acetonitrile, and DMSO, with different boiling points and evaporation kinetics, were used as the organic solvents. HPMCAS solutions in the mentioned solvents demonstrated different viscosities due to different solvent–polymer interactions, as indicated by different solution turbidity. The concentrations of the organic solvent-containing coatings were selected based on desirable flow rates in comparison with the reference Opadry^®^ II coating dispersion. Coatings were applied at the same pan rotation speed, but atomising and pattern air pressure, as well as drying conditions, were different. **Results:** The content of residual solvents in coatings was determined with gas chromatography: low-boiling-point acetone and acetonitrile content was below the LOD, while the content of DMSO, with a boiling point of 189 °C, comprised 1.5 wt.%. A pharmacopoeial approach was utilised to assess uniformity of dosage units via uniformity of content. The accuracy of dosing decreased from acetone- and acetonitrile- to DMSO-based coatings. Because of the high boiling point of DMSO in comparison to ibuprofen, the DMSO-based coating process was the longest, and the amount of ibuprofen loss was the highest. In turn, the precision of dosing via active coating increased from acetone to acetonitrile and to DMSO. The R.S.D. of the uniformity of content decreased along with coating time and fit the power function well (R^2^ = 0.9843). **Conclusions:** Therefore, to answer the main question of this study, proper drug dosing (in terms of accuracy and precision) using drug loading via tablet coating with this specific equipment is possible. Depending on the dose precision desired, the duration of the coating process can vary.

## 1. Introduction

Tablets are the most popular dosage form for oral drug delivery. Drug intake via tablet is non-invasive and does not require specialised medical assistance. Equipment for tablet production is widespread and commonly used [1]. While new technologies such as 3D printing are oriented toward the personalisation of dosage forms [2], “classical” tablet manufacturing can be promptly scaled up to satisfy mass-market demand using comparatively much cheaper technology [1]. Thus, national healthcare systems depend greatly on pharmaceutical dosage forms such as tablets.

To achieve a desirable biopharmaceutical effect, tablets as oral dosage forms are designed so that they can deliver the drug to a specific place within the gastrointestinal tract at a specific drug release rate. This requires achieving a desirable drug absorption rate and pharmacokinetic profile [3,4,5]. Among the tasks involved in tablet design and development are the prevention of undesirable drug–drug interactions for multi-drug products and achieving sequential drug release [6]. Based on the accumulated scientific knowledge, for pharmaceutical pellets (e.g., using a fluid-bed coating) [7], the same tasks can be solved using an active (drug-containing) coating [6,8,9]. So, tablet and pellet coatings can be considered two alternative approaches. Nevertheless, pellets are not applicable for high doses, whereas tablets are. Thus, theoretically, for fixed-dose combinations, where a high dose is in the core and a low dose is in the coating, tablet coating appears to be a more feasible approach.

Tablet coatings are well established in pharmaceutical science and industry for purposes such as enhancing aesthetics, enabling product identification, preventing counterfeiting, masking taste, facilitating swallowing, and achieving delayed or sustained drug release. A list of polymers is widely used for film coating of pellets and tablets and includes organic and aqueous solutions and aqueous polymeric latex dispersions [10,11,12]. The same polymers could be used to obtain an amorphous solid dispersion of a drug, which is the contemporary approach used to increase the apparent solubility and improve the oral bioavailability of poorly soluble drugs. Polymer-based amorphous solid dispersions prepared by spray-drying are well known within the scientific community [13], whereas preparation by fluid-bed coating is a lot less popular [14]. Examples of active coating and amorphous active coating of tablets are rarely seen in the scientific literature [13,15]. Compression-sensitive drug properties, low doses, and the requirement for handling in the containment area are additional reasons to consider active coating [16].

One of the principal limitations of drug-coated tablets is inherently linked to the surface area available for coating. Unlike multiparticulate systems such as pellets, tablets offer a limited surface area, restricting the maximum amount of the drug that can feasibly be incorporated into the coating layer [17]. This physical constraint directly translates into a limit on drug loading capacity in the coating, often restricting the API content to relatively low percentages. Thus, high drug doses pose a particular challenge for drug-coated tablets, as the maximum feasible coating thickness is governed by processability and mechanical robustness considerations. Thicker coatings require longer processing times and could negatively impact manufacturing throughput.

The variability in tablet coatings in pan coaters during the coating process, including coating weight gain and coating thickness, is a current practical issue in pharmaceutical formulation and manufacturing [16,18,19,20,21,22,23,24,25,26,27,28,29]. A lot of effort is currently spent on controlling and monitoring these coating variables, including process analytical technologies [30,31,32,33]. Apart from practical experiments, scientists nowadays additionally use computational fluid dynamics, the discrete element method, and Monte Carlo simulation methods [34,35,36,37,38].

Active coating for tablets in pan coaters and pellet coating in fluid-bed coaters represent the two known strategies in pharmaceutical formulation. However, despite their shared goals, significant differences exist between these two approaches in terms of maturity and process optimisation. For tablet coating, there is a heavy dependence on empirical adjustments tailored to specific machines or products rather than robust, predictive models [35,39]. This lack of a mechanistic understanding in active tablet coating leads to challenges in scaling up and reproducing coating quality, consistency, and functionality. Factors such as uniform distribution of drug–polymer layers, coating thickness, and adhesion are influenced by numerous interdependent parameters whose effects are not thoroughly quantified for tablets [13]. Formulating coatings for tablets loaded with drugs, polymers, and an organic solvent faces significant challenges due to solvent–polymer–drug interactions and plasticisation effects [40].

The mechanistic understanding linked to solvent evaporation kinetics remains largely unexplored in active tablet coating. As a result, formulations for tablets frequently rely on trial-and-error screening rather than rational solvent and polymer selection based on solid scientific principles. The physical robustness of tablet coatings is harder to predict given their non-uniform thickness and susceptibility to moisture-induced plasticisation during drying. Polymer selection for coatings that balances adhesion, mechanical integrity, and physical stability under industrial conditions is still underdeveloped for tablets compared to pellet coatings [41]. Despite extensive industry usage, tablet film coating remains a largely empirical process with limited scientific models to guide scale-up or cross-platform transfer.

Considering the possibility of physical separation of drugs in the tablet, achieving sequential drug release, increasing apparent solubility, and improving the oral bioavailability of poorly soluble drugs, we have raised practical questions in this work. Can we properly dose drugs by drug loading via tablet coating with a specific coater? Under what conditions can tablets be coated, and what dose precision and accuracy can be achieved? This work aimed to investigate the drug content uniformity of placebo tablets coated with drug–polymer solutions using the easily accessible model drug ibuprofen (with a boiling point of 157 °C) and organic solvents with different boiling points. The novelty of this work lies in probing the possibility of actively coating the drug–polymer solid dispersion on the tablet cores using a set of organic solvents with a widely used laboratory coating machine. The results of this paper are going to facilitate the estimation of dosing accuracy and precision for other experiments conducted with similar equipment.

## 2. Materials and Methods

Ibuprofen (BASF SE; Ludwigshafen, Germany); acetate succinate (HPMCAS; “L” grade; Shin-Etsu Chemical Co., Ltd., the Tokyo, Japan); Opadry^®^ II (85F240326 pink; Colorcon, Dartford, UK); and the quality of organic solvents, namely acetone, dimethyl sulfoxide (DMSO), acetonitrile, methanol (Merck KGaA, Darmstadt, Germany), and dimethylformamide (DMF; Thermo Scientific, Darmstadt, Germany), complied with the pharmacopoeia requirements.

### 2.1. Preparation of Drug–Polymer Solutions in Organic Solvents for Coatings

Solutions of IBU:HPMCAS–solvent were prepared using each solvent (acetone, acetonitrile, and DMSO) at a constant drug–polymer ratio of 25:75 (*w*/*w*) and amount but with different amounts of solvents (Table 1). Ibuprofen was dissolved in each organic solvent using a magnetic stirrer. Afterwards, the polymer was added during mixing, and mixing was continued until the complete dissolution of clumps. The solutions were left to stand overnight before testing.

### 2.2. Flow Rate and Kinematic Viscosity of Solutions

For the flow rate and kinematic viscosity tests, ibuprofen was not added to the solution. The flow rate and kinematic viscosity of the polymer–solvent solutions (100 mL, 7.5% *w*/*v*; and two additional concentrations, 5% and 15% *w*/*v*, for flow rate) were studied using a viscosity cup with an orifice diameter of 4 mm according to DIN 53211 (TQC Sheen; LL Capelle aan den IJssel, The Netherlands) [42].

The cup was positioned vertically and filled with 100 mL of the polymer–solvent solution. The time and mass of liquid release were measured (n = 3). The flow rate was calculated as the ratio of the solution mass (g) to the time (*t*, s) required for the solution to flow out of the cup (g/s). Kinematic viscosity (*v*, mm^2^/s) was calculated according to the following equation [39]:

$$v\;=\;4.57\times t\;-\;\frac{452}{t}$$

The DIN equation was derived based on the kinematic viscosity measurement of standard solutions. Thus, this equation is applicable only for liquids with a volume of 100 mL that flow out through the cup’s orifice within the time range from 25 to 150 s [42].

### 2.3. Turbidity Measurement

For turbidity measurements, ibuprofen was not added to the solutions. The solutions of HPMCAS and solvent (7.5% *w*/*v*) were prepared in acetone, acetonitrile, and DMSO. The UV-Vis spectra (n = 3) were collected in the range of 190–1100 nm in a 10 mm cuvette by a UV-Vis spectrophotometer (UV-1900i; Shimadzu, Kyoto, Japan).

### 2.4. Thermogravimetric Analysis (TGA)

The evaporation rate from the polymer–solvent samples was examined using a Thermal Advantage Q50 TGA (TA Instruments, New Castle, DE, USA). Samples (approximately 10 mg) were heated in an open aluminium pan at a constant temperature of 80 °C/min. Nitrogen at a flow rate of 50 mL/min was used as the purge gas for all TGA experiments. Data was processed with a Universal V4.5A software (TA Instruments, USA). The tests were repeated in triplicate.

### 2.5. Placebo Tablet Preparation

The placebo tablets were prepared from a microcrystalline cellulose (49.5 wt.%), anhydrous lactose (49.5 wt.%), and magnesium stearate (1.0 wt.%) powder mixture. To obtain biconvex tablets (with a diameter of 13 mm) with a weight of 660 mg, round concave (with a hemisphere radius of 6.5 mm) punch-die sets, a rotary tablet press, and a forced powder filler were utilised (model XL 100; Korsch AG, Berlin, Germany), with a constant tableting speed (turret speed of 40 rpm) and feed frame forced die filling speed (with a rectangular straight paddle geometry) of 20 rpm [43].

### 2.6. Surface Area and Specific Surface Area of Tablets

The surface area (*SA*) of the tablets was calculated based on the following equations [44]:

$$SA=2\;\cdot \;\pi \;\cdot \;\left(r\cdot B+r^{2}+h_{C}^{2}\right)\;\mathrm{and}\;h_{C}^{2}=\;\frac{H-B}{2}$$

where *r* is the radius of the tablet; *B* is the band height; *h_C_* is the height of the curvature; and *H* is the overall height.

In turn, specific *SA* (*SSA*) was calculated based on the average *SA* and average mass (*m*) of the tablets by the following equation:

$$SSA=\frac{SA}{m}$$

### 2.7. Tablet Coating

The placebo tablets were coated using a drum coater (GC1; Glatt GmbH, Binzen, Germany) with one nozzle (970/0 S75 ABC-Technology^®^; Düsen-Schlick GmbH, Coburg, Germany) and under the conditions/parameters shown in Table 1. Despite the detailed description of the tablet coating parameters, it should be mentioned that in this study, the experimental coating conditions were not optimised.

### 2.8. Gas Chromatography (GC) Method for Residual Solvent Quantification

Residual solvent analyses were performed on a Nexis GC-2030 GC with a flame ionisation detector (FID) and an automated headspace sampler (HS-20NX) from Shimadzu Corporation (Tokyo, Japan). The system was controlled by a LabSolutions Chromatography System. All solvents were separated on an Rxi-5 ms (30 m × 0.32 mm, 0.25 µm film thickness) capillary column from Restek (Bellefonte, PA, USA). The GC-FID experimental conditions are detailed in Table 2.

A volume of 0.25 mL of each solvent (acetonitrile, acetone) was transferred into a 25 mL volumetric flask, previously filled with about 3 mL of DMF, diluted with DMF to volume, and mixed. This was a standard stock solution A. A volume of 0.5 mL of each solvent (acetonitrile, acetone) was transferred into a 10 mL volumetric flask, diluted with DMF to volume, and mixed (standard stock solution B). Then, 0.5 mL of standard stock solution (A or B) was transferred into an appropriate headspace vial containing 2.5 mL of water. The stopper and cap were applied; all vials were vortexed. From the prepared standard stock solution A, serial dilutions were performed to prepare the linearity standards (0.75, 0.25%, 0,10%, and 0.05%). A volume of 0.5 mL DMF with 2.5 mL water was applied as a blank.

For sample analysis, tablet coatings were weighed and diluted at a concentration of 55 mg of separated coating per mL. A volume of 0.5 mL of each solution of tablet coatings was transferred into 10 mL headspace vials containing 2.5 mL water. System suitability was proved by mixing 0.5 mL of tablet coating solution with 0.5 mL of the appropriate solvent standard stock solution. A volume of 1.0 mL of this solution was transferred to an appropriate headspace vial containing 2.0 mL of water. The stopper and cap were applied, and solutions were vortexed.

DMSO is known to coelute with DMF; therefore, we changed the solution solvent (diluent) from DMF to methanol. The DMSO coating was dissolved in methanol. The DMSO standard solution was prepared at a concentration of 1.2 mg/mL (29 mg in 25 mL methanol) and 22.4 mg/mL (560 mg in 25 mL methanol), vortexed for a while, and shaken well. Then, these DMSO standard solutions were diluted to 3.7, 1.2, 0.2, 0.1, and 0.04 mg/mL. A total of 3.0 mL of each DMSO standard solution was transferred to an appropriate headspace vial. The stopper and cap were applied, and solutions were vortexed.

DMSO content in the tablet coating in terms of wt.% or ppm was calculated according to the equations below.

$$DMSO\;\left(\%\right)=\frac{sample\;peak\;area}{standard\;peak\;area}*standard\;concentration\;\left(\frac{mg}{mL}\right)*\frac{dilution\;(mL)}{sample\;weight\;\left(mg\right)}*100\%$$

$$DDMSO\;\left(ppm\right)=\frac{sample\;peak\;area}{standard\;peak\;area}*standard\;concentration\;\left(\frac{mg}{mL}\right)*\frac{dilution\;(mL)}{sample\;weight\;\left(mg\right)}*10^{6}$$

The tests were repeated in triplicate for each sample.

### 2.9. Determination of Drug Content in Tablets

HPLC-UV was carried out using a Vanquish HPLC (Thermo Scientific, Dionex Softron GmbH, Germering, Germany) equipped with a quaternary pump and an autosampler, and a UV-Vis Detector was used to determine the content of ibuprofen in the tablets (n = 10 for each tablet batch). The tablets were sonicated with acetonitrile (6% of total volume of volumetric flask) for 15 min, cooled down to room temperature, filled up to volume with water at pH 2.5 (adjusted with phosphoric acid), and mixed and filtered with a 0.45 µm hydrophilic PVDF membrane filter (Merck Millipore Limited, Carrigtwohill, Ireland) prior to placement in the HPLC vial. Separation was performed using a Zorbax Eclipse Plus C18 (150 × 2.1 mm, 5 μm) analytical column from Agilent (Santa Clara, CA, USA), with a SecurityGuard C-18 (4 × 3 mm; Phenomenex, Torrance, CA, USA) guard column. The mobile phase consisted of water with a pH of 2.5 (adjusted with phosphoric acid) and acetonitrile (40:60, *v*/*v*). The flow rate of the mobile phase was 0.28 mL/min in the isocratic elution mode. A sample volume of 5 μL was injected into the column at a constant temperature of 30 °C, with the run time set to 8 min. The UV wavelength λ was 221 nm. Data analysis of the obtained chromatograms was carried with the Chromeleon software (version 7.3.1).

### 2.10. Uniformity of Dosage Forms: Test for the Uniformity of Content

The uniformity of dosage units was performed considering the requirement described in the current version of the European Pharmacopoeia (chapter 2.9.40) [45]. The uniformity of content was evaluated based on the acceptance value (*AV*). *AV* was calculated using the following equation:

$$AV=\;\left|M-\;\bar{X}\right|+k\;\cdot \;s$$

*AV*—acceptance value; *M*—labelled content of active substance; $\bar{X}$—mean value; *k*—acceptability constant (*k* = 2.4 because the number of tablets was equal to 10); *s*—sample standard deviation. In our research, to compare the results for different tablet batches, the following assumption was made: $M=\bar{X}$.

### 2.11. Drug–Polymer Film Behaviour in Different Media

A 0.1 N solution of HCl with a pH of 1.2 (simulating the pH of a fasting stomach) and a phosphate buffer solution with a pH of 6.8 (simulating the pH of an intestinal medium) were prepared. The tablets’ polymer-based coating was carefully peeled off. A volumetric flask was filled with 25 mL of the respective media. The drug–polymer-based film prepared using acetonitrile (with an exact weight of approx. 30 mg) with an ibuprofen content of approx. 7.5 mg was added. The volumetric flask was mixed with a magnetic stirrer at room temperature, and every 5 min, UV absorption at λ_max_ of 221 nm was monitored with a UV spectrometer (UV-1900i; Shimadzu Corporation, Japan). The proportions of the drug and media were proportional to an ibuprofen dose of 300 mg per 1 L of dissolution media. The tests were repeated in triplicate.

## 3. Results and Discussion

Most drugs with poor aqueous solubility, such as ibuprofen and others, are soluble in organic solvents. Most pharmaceutically applicable polymers, including polymers for the preparation of amorphous solid dispersions, are soluble in organic solvents as well. Thus, organic solvents are widely used in spray-drying, fluid-bed, and tablet coating processes. In our experiment, ibuprofen and HPMCAS were used as a model drug and polymer in a proportion of 25:75 (*w*/*w*). The drug and polymer were dissolved in the following organic solvents: acetone, acetonitrile, and DMSO.

**Figure 1:**
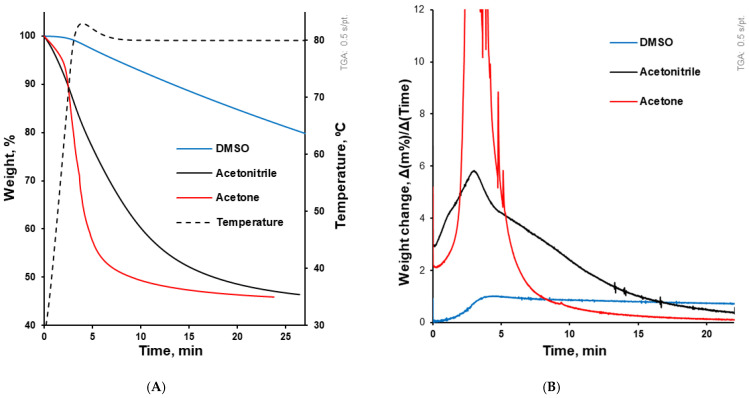
(**A**) Solvent evaporation kinetics from solvent–polymer solution upon testing in the DSC pan. The dotted line (related to the right axis) illustrates the temperature course, while the other three lines illustrate the evaporation kinetics of solvents from the solvent–polymer solution. (**B**) This graph accompanies (**A**) and illustrates solvent evaporation in terms of weight change per time (dm/dt).

As they are dependent on molecular weight and structure, the boiling points of acetone, acetonitrile, and DMSO are 56, 82 and 189 °C, respectively [46]. These organic solvents demonstrated different evaporation rates (Figure 1) from the solvent–polymer solutions. In accordance with their boiling points, the evaporation rate from 20% (*w*/*v*) HPMCAS solution decreased from acetone to acetonitrile and to DMSO. Thus, the coating solutions based on these solvents required different coating regimes to be evaporated during the coating process.

**Figure 2:**
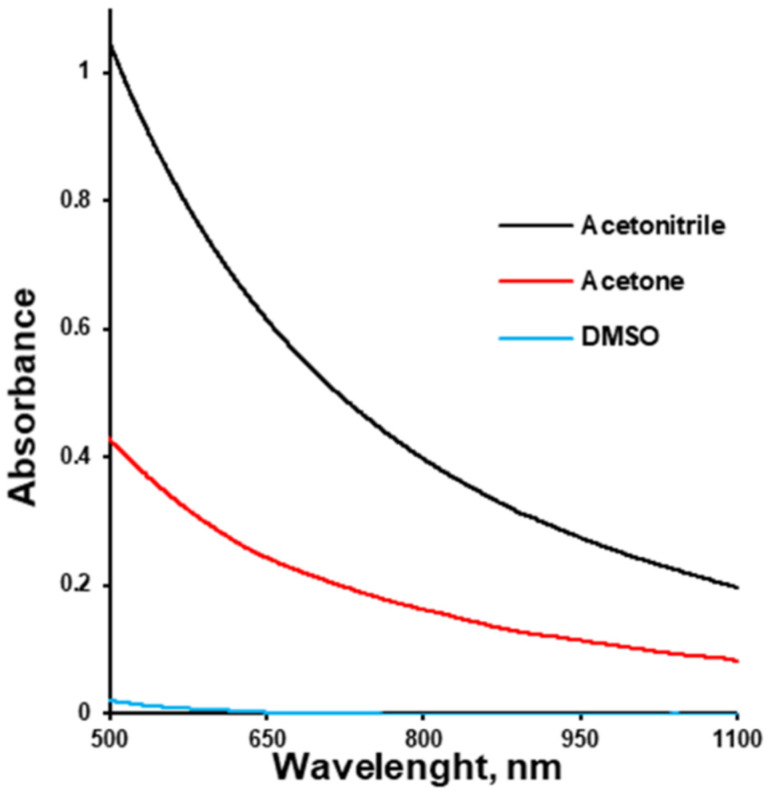
Turbidity of HPMC-AS solution (7.5% *w*/*v*) depending on the solvent.

On the other hand, drug–polymer solutions should have appropriate viscosities in order to be properly sprayed during tablet film-coating. The kinematic viscosity of the polymer–solvent solutions at a concentration of 7.5% (*w*/*v*) increased from acetone to acetonitrile to DMSO and amounted to 94.38 ± 4.99, 94.40 ± 2.51, and 227.21 ± 2.13 mm^2^/s. It should be mentioned that the viscosity values of the pure solvents were 0.3, 0.3, and 2.5 mPa∙s, respectively [46]. Thus, the difference in viscosity between solutions of the same concentration in different solvents can be attributed to different solvent–polymer interactions. These different interactions were indirectly illustrated by the polymer solutions’ turbidity (Figure 2). DMSO ensured a transparent HPMCAS solution, while the polymer solution in acetone was turbid, and that in acetonitrile was even more turbid.

Turbidity is an optical property that quantifies the cloudiness or haziness of a fluid, caused primarily by the scattering of light due to suspended, aggregated, or phase-separated domains. Within polymer solutions, turbidity serves as an indirect but informative indicator of the extent of heterogeneity in the system. Specifically, in polymer solutions, the onset and degree of turbidity correlate with micron- to nanoscale structural heterogeneity emerging from polymer–solvent interactions [6]. Polymer–solvent interaction influences the polymer chain conformation in solution, which ranges from fully relaxed coils in good solvents to tightly coiled or collapsed states in poor solvents. In good solvents exhibiting strong polymer–solvent affinity, polymer chains assume relaxed, expanded conformations, which often correlate with higher solution viscosities and homogeneity. This conformation facilitates the formation of uniform films with minimal phase separation and low turbidity [7,8]. Lower turbidity and higher viscosity are indicators of higher polymer solvation (opening), which results in higher mechanical strength of the polymeric coating due to the higher entanglement of polymeric chains.

**Figure 3:**
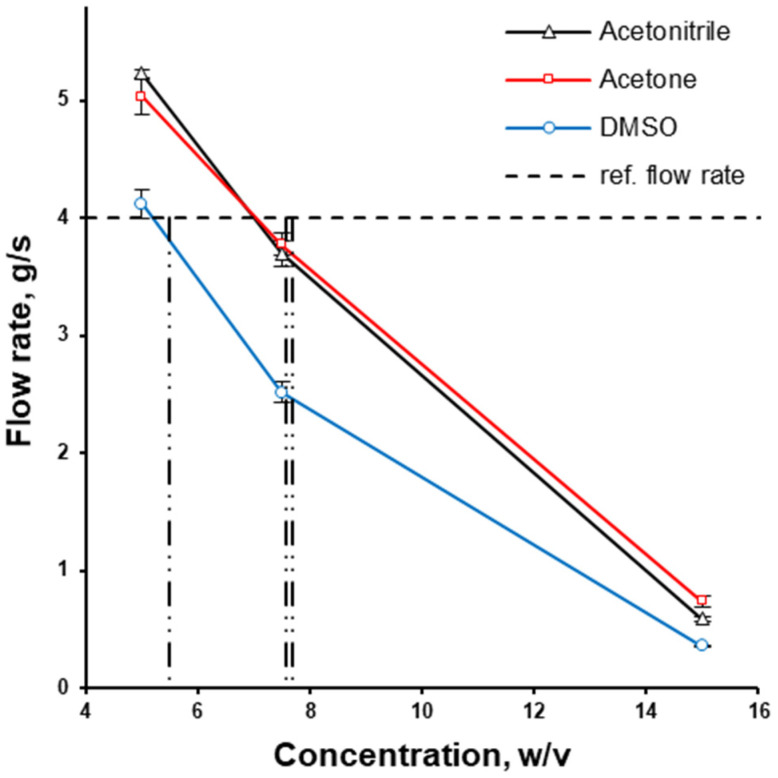
The effect of solvent type and concentration on the flow rate of HPMCAS solution; the utilised concentrations of HPMCAS solutions in organic solvents are depicted with vertical lines.

The kinematic viscosity of some solutions with the concentration of interest was outside the calibration range of the cup used. Therefore, the same cup used for the kinematic viscosity measurement was used to compare the flow rate (g/s) of polymer solutions at different concentrations. The flow rate decreased as the concentration increased. At every concentration in the range between 5 and 15% of HPMCAS (*w*/*v*), the flow rate of DMSO was the lowest out of the three solvents used, while the flow rates of acetone solutions were almost the same as the flow rate of acetonitrile (Figure 3). The aqueous-based coating dispersion Opadry^®^ II at a concentration of 20% (*w*/*v*) was used as a market reference ensuring a non-problematic coating process and thus possessing an appropriate viscosity. The flow rate of Opadry^®^ II aqueous dispersion at 20% *w*/*v* was 4.0 g/s. This is why HPMCAS solutions in organic solvents were chosen at concentrations of 7.57%, 7.68%, and 5.55% *w*/*v* for acetone, acetonitrile and DMSO, respectively, to obtain a close-to-desirable flow rate (Figure 3).

Using the same drug–polymer proportion, the same amount of ibuprofen (to achieve a target dose of 12.63 mg/tablet) was coated from different organic solutions onto the same amount of placebo tablets (350 g; proportional to 530 tablets). The surface area of the average tablet in this experiment comprised around 510 mm^2^, and the corresponding specific surface area was 6.1 cm^2^/g. For the purposes of comparison, it can be mentioned that the specific surface area of a microcrystalline cellulose sphere with a 300 µm diameter comprises 137 cm^2^/g [17], which illustrates a difference in SSA of more than one order of magnitude.

**Figure 4:**
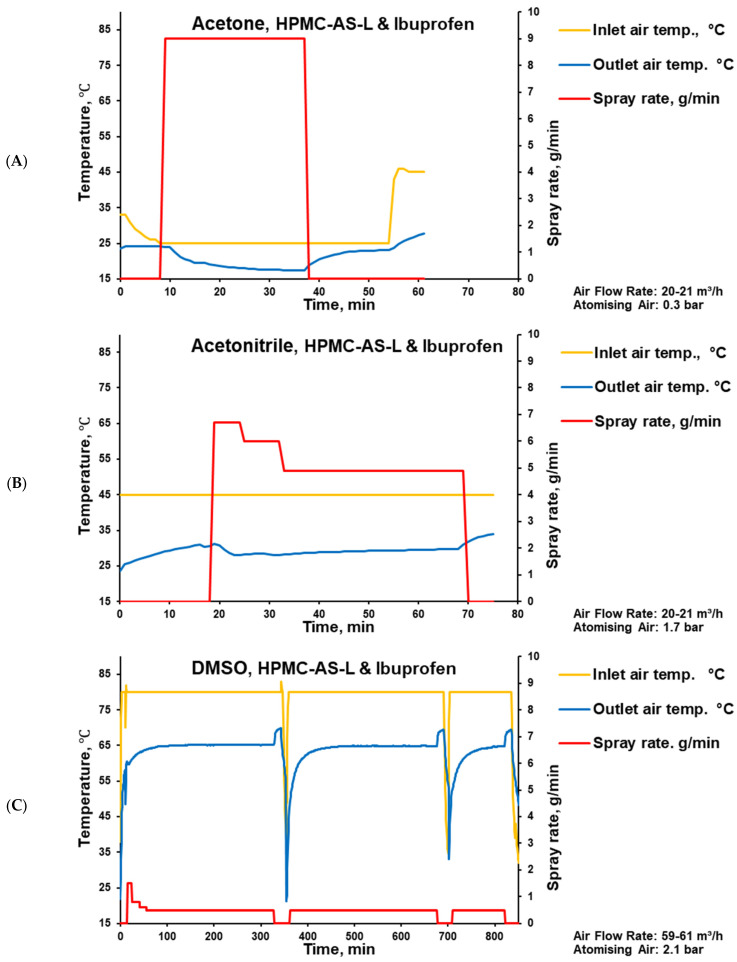
Parameters of placebo tablet coating process with HPMC-AS-L-based coating and ibuprofen using acetone (**A**), acetonitrile (**B**), and DMSO (**C**) as solvents.

Tablets were coated in a state-of-the-art perforated pan coater, which, based on the batch size, can be considered laboratory-size. The coater was used with a single two-component nozzle, a perforated pan width of 11 cm, and an internal diameter of 30 cm. In accordance with the solvents’ boiling points, the coating time increased from 28 min for acetone (Figure 4A) to 50 min for acetonitrile (Figure 4B) and 739 min for DMSO-containing coatings (Figure 4C). Considering a pan rotation speed of 12 rpm, these coating times correspond to 336, 600, and 8868 rotations, respectively (Table 1). Exemplified with tablets coated using acetonitrile and acetone, the resulting coating thickness (separated from the core and measured with a calliper) was about 100 μm.

**Figure 5:**
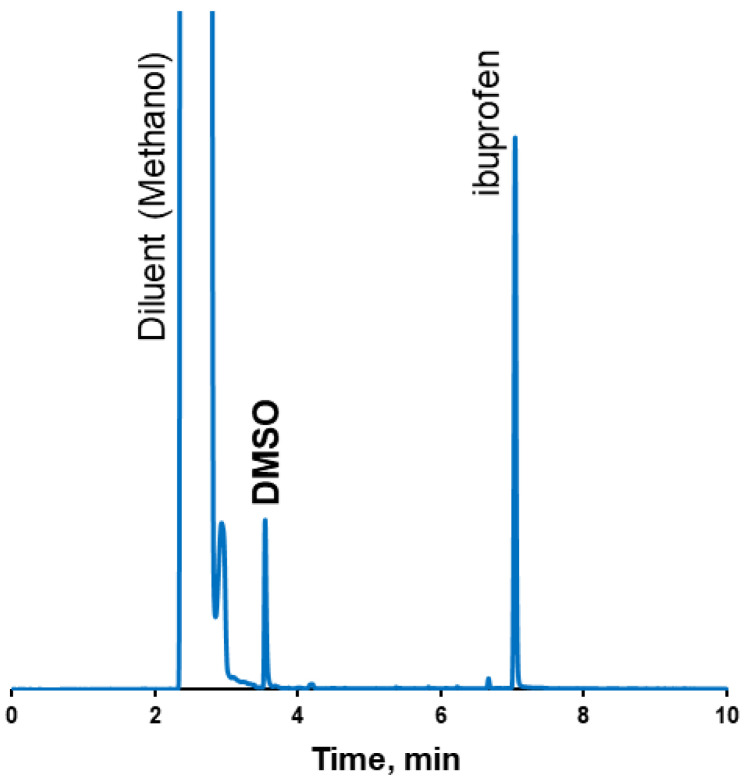
GC-FID chromatogram of tablet coatings from DMSO.

The boiling point of ibuprofen (157 °C) is lower than the boiling point of DMSO (189 °C). Because of the relatively low boiling point of ibuprofen, the thermal methods for the determination of the residual solvents, such as TGA, were not applicable. Using gas chromatography, acetone and acetonitrile were found in amounts below detectability limits, while the determined DMSO content comprised 1.5 wt.% of the coating (Figure 5, Table 3). It should be mentioned that, according to the ICH Q3C acceptance criteria, DMSO is a solvent classified as class 3—low toxic potential. And the upper consumption limit of class 3 residual solvents comprises 50 mg per day [47].

For the DMSO-based coating, even with a relatively low spray rate and high drying temperature (Figure 4), hints of sticking were observed during the coating process. These observations are in line with the high residual solvent content determined in the respective coating, because DMSO has the slowest evaporation rate (the highest boiling point). This can be explained by the decreased glass transition temperature (Tg) of the coating (drug–polymer–solvent system), which can trigger tablet surface sticking.

Drug content uniformity is predetermined by the pharmacopoeia, and it is an important quality parameter for the assessment of dosage form. The accuracy of dosing can be defined as the difference between the target (labelled) and the mean determined dose, while the precision of the dose can be described with R.S.D. In our study, we used non-optimised coating parameters and obtained different drug loads, which is obvious from the mean value ($\bar{X}$) of ibuprofen (mg) per tablet (Table 4). Drug content in the coated tablets increased from the DMSO-based (6.07 ± 0.18 mg/tab.) to the acetonitrile-based (12.10 ± 0.53 mg/tab.) and to the acetone-based (12.38 ± 0.65 mg/tab.) coating. The difference in drug content between the acetonitrile- and acetone-based coatings was not significant, considering the ANOVA single-factor analysis resulted in a *p*-value of 0.32. Meanwhile, the low ibuprofen content in DMSO-based coatings can be explained by the high drying inlet air temperature (80 °C) and ibuprofen evaporation.

The accuracy of dosing increased from DMSO to acetonitrile and to acetone, supported with average values of 6.07 (unsatisfactory), 12.10, and 12.38, respectively (Table 4), in comparison with the target drug loading of 12.63 mg/tab (Table 1). The losses were mainly associated with spray-drying losses to exhaust air, because no visible losses were observed on the drum surface. All the results for the tablet doses regarding acetone and acetonitrile solvents complied with the pharmacopoeia acceptance values and the maximum allowed values (Table 4) [45]. It should be mentioned that ibuprofen has been selected as a pure model drug, with the shortcoming that, in the case of DMSO, the accuracy of the dosing was unsatisfactory because of ibuprofen evaporation, whereas this part of the study was meant to investigate the effect of prolonged coating times on precision.

**Figure 6:**
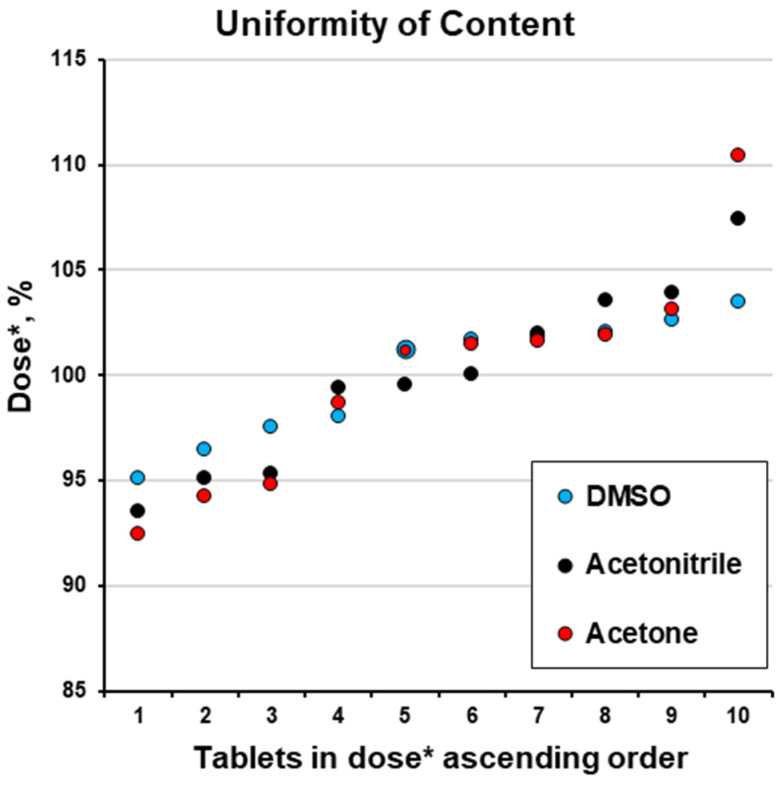
Uniformity of content (dose *, %; please see Table 4).

To make these results comparable, we accepted the obtained mean value ($\bar{X}$) as the target dose (*M*) and expressed the values for each tablet’s content as a percentage of the target dose (dose *, %; Table 4). This allowed us to compare the doses for batches coated with solutions based on different organic solvents. This approach is not practical; in practice, a specific weight gain and dose should be achieved. This is because in serial industrial production, a specific weight of the dosage form is correlated with a specific dose of the pharmacologically active drug. Nevertheless, this approach was used because, in accordance with the scope of this study, it allowed us to compare non-optimised formulations/coating conditions. So, considering the assumption of ($M=\bar{X}$), except for one tablet using the acetone solvent, all the results are within a narrow range of 90 and 110% (Figure 6).

**Figure 7:**
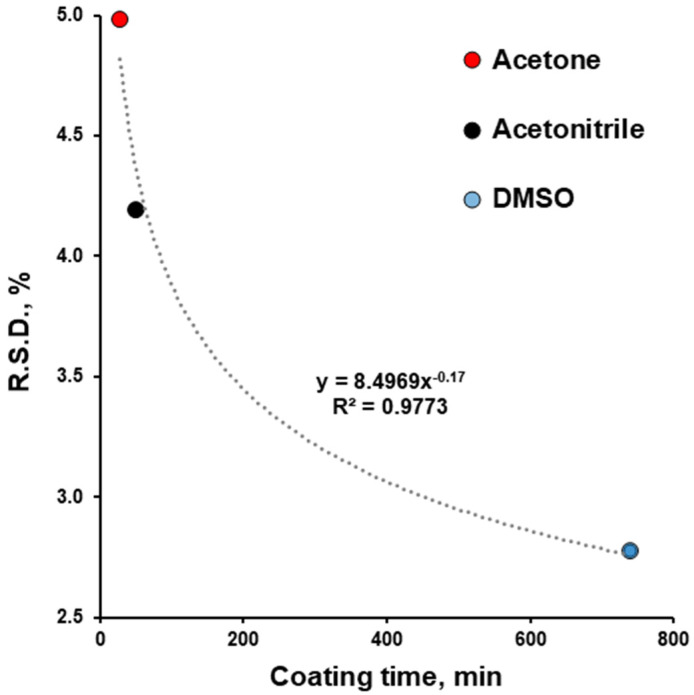
Effect of coating time on relative standard deviation (R.S.D.) of content of uniformity of film-coated tablets.

The precision of ibuprofen dosing increased from acetone to acetonitrile and to DMSO. The increase in coating time from 28 to 739 min improved (decreased) the R.S.D. of dosing from 5.25 to 2.93% (Figure 7). This finding agrees with previously reported information [16,21,25]. The R.S.D. for the uniformity of content time and its good fit to the power function (R^2^ = 0.9843) are in agreement with previously reported information [25,38]. So, for subsequent experiments with the same equipment and for scale-up, the coating duration time in combination with the spray rate can be considered to influence dosing precision.

The limitations of the active coating approach in terms of practically applied doses are obvious from the example of the model drug used. As a single dose of ibuprofen is 200 mg, a patient would need to take 16 tablets (in the case of acetone-coated tablets, as each contains 12.5 mg of ibuprofen; Table 4) for one therapeutic dose. This clearly illustrates the limitation in the applicability of this method for relatively low-dose drugs.

This study was not aimed at investigating drug release from coated tablets; nevertheless, a short experiment was performed to clarify what could be expected. Based on the properties of the drug (a weak acid) and the polymer (which contains functional groups with properties of a weak acid, with an opening pH of around 5.5), the resulting drug–polymer coating was expected to be dissolved in the intestine. Our observations of tablet behaviour in a 0.1 N solution of HCl with a pH of 1.2 (simulating the pH of a fasting stomach) and in a phosphate buffer solution with a pH of 6.8 (simulating the pH of an intestinal medium) confirmed no drug release in pH 1.2 and fast (in approx. 15 min) release in pH 6.8.

## 4. Conclusions

An ibuprofen–HPMCAS (25:75, *w*/*w*) active coating was applied to approximately 530 tablets using acetone, acetonitrile, and DMSO as organic solvents and using a laboratory-scale coating machine (GC1; Glatt GmbH, Germany) with a perforated pan width of 11 cm, an internal diameter of 30 cm, and a single two-component Schlick nozzle (970/0 S75 ABC-Technology^®^). The coating conditions and parameters were well documented, making them applicable for computational model validation. Additionally, this information could be useful for laboratory- or pilot-scale coatings at the early stages of drug products development and scale-up. The choice of solvent is often dictated by the solubility of drugs and polymers in them; however, this study highlights the importance of solvent properties, such as boiling point, in determining process duration, drug content (for drugs with a relatively low boiling point), and drug content uniformity. Because ibuprofen’s boiling point is lower than that of DMSO, approximately half of the drug’s amount was lost during the drying process, and dosing accuracy for this coating was unsatisfactory. The dosing accuracy for acetone- and acetonitrile-based coatings complied with pharmacopeial requirements in terms of the uniformity of dosage forms estimated by the uniformity of content. Dosing precision improved with the increase in coating time (or pan rotation). R.S.D. amounted to 4.42, 5.25, and 2.93 for 50, 28, and 739 min of coating time (or for 600, 336, and 8868 pan rotations), respectively. The dependence of R.S.D. for the uniformity of content on coating time has a good fit to the power function (R^2^ = 0.9843). This information can be used for the estimation of required coating times to achieve the desired uniformity of drug content. Therefore, to answer the main question of this study, proper drug dosing (in terms of accuracy and precision) via drug loading using tablet coating with this specific equipment is possible. Depending on the dose precision desired, the duration of the coating process can vary.

## Figures and Tables

**Table 1:** Coating parameter of MCC placebo tablets by ibuprofen–HMPCAS solution.

	Pan Characteristics		
**Pan type**	Fully perforated side-vented coating pan		
**Pan diameter, cm**	30		
**Pan width, cm**	11		
	**Coating Composition**		
**Solvent**	acetone	acetonitrile	DMSO
**Drug-to-polymer ratio, *w*/*w***	25:75	25:75	25:75
**Solid content, % *w*/*v***	10.09	10.24	7.40
**Polymer content, % *w*/*v***	7.57	7.68	5.55
**Volume of coating solution, mL**	278.6	274.7	380.3
	**Run Objectives**		
**Theoretical weight gain, wt.%**	7.66		
**Theoretical drug load, wt.%**	1.91		
**Theoretical drug load, mg/tab.**	12.63		
**Tablet charge, g**	350		
**Single tablet weight (Av.), mg**	660		
**Tablet shape and size**	Biconvex, D = 13 mm, hemisphere r = 6.5 mm, H = 6 mm		
	**Gun Configuration and Settings**		
**Number of guns**	1		
**Gun type**	Schlick nozzle (970/0 S75 ABC-Technology^®^)		
**Spray pattern**	Oval flat spray pattern		
**Nozzle diameter, mm**	0.8	0.8	0.8
**Gun to bed distance, cm**	18	18	18
**Atomized air pressure, bar**	0.3	1.7	2.1
**Pattern air pressure, bar**	0	0	1.4
	**Other Process Parameters**		
**Pan speed, rpm**	12	12	12
**Intel air flow rate, m^3^/h**	20.5	20.8	60
**Intel air temperature, °C**	25	45	80
**Outlet air temperature, °C**	24–17	30	60–65
**Spray rate, g/min**	9.0	6.7–4.9	0.5
**Coating time, min**	28	50	739

**Table 2:** GC-FID operation conditions for residual solvent analysis.

	Setting for Residual Solvents	
Parameters	Acetonitrile or Acetone	DMSO
***for HS-20NX***		
Oven	80 °C	
Sample line temperature	180 °C	
Transfer line temperature	180 °C	
Precondition and time	Shaking for 60 min	Shaking for 20 min
Run time	45 min	10 min
***for Nexis GC-2030***		
Column	Restek Rxi-5 ms (30 m × 0.32 mm, 0.25 µm film thickness column).	
Injector	200 °C, split ratio 1:10 (splitless)	250 °C, splitless
Detector	FID, 250 °C	
Carrier gas	constant flow of helium (He) at 1 mL/min	
Thermal conditions of column	Initial isothermal temperature hold at 40 °C for 20 min; followed by temperature increase 10 °C/min up to 250 °C	Initial isothermal temperature hold at 70 °C for 1 min; temperature increase of 50 °C/min up to 130 °C; isothermal temperature hold at 130 °C for 1 min; followed by temperature increase of 50 °C/min up to 220 °C; and isothermal hold at 220 °C for 5 min

**Table 3:** Table summarising results of residual solvents in tablet coatings.

	Solvents in Tablet Coating					
	DMSO		Acetonitrile		Acetone	
	Sample Solution	In Coating	Sample Solution	In Coating	Sample Solution	In Coating
Units	ppm	wt. %	ppm	wt. %	ppm	wt. %
run #1	13,469	1.3	ND	ND	ND	ND
run #2	16,953	1.7	ND	ND	ND	ND
run #3	15,224	1.5	ND	ND	ND	ND
Av.	15,215	1.5	ND	ND	ND	ND
S.D.	1742	0.2	ND	ND	ND	ND
***Method-related information***						
Retention time, min	3.5 min		2.9 min		2.9 min	
LOQ concentration	0.038 mg/mL (38 ppm)		0.02 mg/mL (20 ppm)		0.03 mg/mL (30 ppm)	
LOD concentration	0.013 mg/mL (13 ppm)		0.007 mg/mL (7 ppm)		0.01 mg/mL (10 ppm)	
Linearity range	0.038–1.15 mg/mL 38–1493 ppm		0.04–0.5 mg/mL 40–494 ppm		0.05–0.6 mg/mL 50–589 ppm	
Recovery, %	98.2		101.8		103.2	
Calibration eq.	Peak area = 69.7 × 10^4^·C_DMSO_ − 9.98 × 10^4^		Peak area = 2 × 10^6^·C_ACN_ − 4 × 10^6^		Peak area = 2 × 10^7^·C_Acetone_ − 3 × 10^6^	
Regression Coeff. (R^2^)	0.997		0.986		0.991	

ND—not detected.

**Table 4:** Uniformity of content (Ph. Eur. [45]).

	Determined Values			$\mathrm{Assumption}:\;M=\bar{X}$					
	DMSO	Acetonitrile	Acetone	DMSO		Acetonitrile		Acetone	
	mg/tabl	mg/tabl	mg/tabl	% of Dose *	Dev. ^a^	% of Dose *	Dev.	% of Dose *	Dev.
Tablet #1	6.17	12.53	12.21	101.7	1.7	103.6	3.6	98.7	1.3
Tablet #2	5.77	12.11	12.56	95.1	4.9	100.1	0.1	101.5	1.5
Tablet #3	5.86	11.53	12.58	96.5	3.5	95.3	4.7	101.6	1.6
Tablet #4	6.23	12.03	11.44	102.6	2.6	99.4	0.6	92.4	7.6
Tablet #5	6.28	12.57	11.74	103.5	3.5	103.9	3.9	94.8	5.2
Tablet #6	6.18	12.05	11.66	101.9	1.9	99.6	0.4	94.2	5.8
Tablet #7	6.20	12.34	12.76	102.0	2.0	102.0	2.0	103.1	3.1
Tablet #8	5.95	11.32	13.67	98.0	2.0	93.6	6.4	110.5	10.5
Tablet #9	6.15	13.00	12.53	101.2	1.2	107.5	7.5	101.2	1.2
Tablet #10	5.92	11.51	12.61	97.6	2.4	95.1	4.9	101.9	1.9
**Mean value (** $\bar{X}$ **)**	6.07	12.10	12.38	100.0	–	100.0	–	100.0	–
**Target dose (*M*)**	12.63	12.63	12.63	–	–	–	–	–	–
**Sample S.D. (*s*)**	0.18	0.53	0.65	2.93	–	4.42	–	5.25	–
**R.S.D., %**	2.93	4.42	5.25	**2.93**	–	**4.42**	–	**5.25**	–
$AV=\left|M-\bar{X}\right|+k\cdot s$	–	–	–	**7.03**	*passed*	**10.60**	*passed*	**12.60**	*passed*
**Max allowed *AV = 15%* (or 1.90 mg)** **^b^**	*did not pass* *51.9% > 15%*	*Passed* *4.2% < 15%*	*passed* *2.0% < 15%*	**15.00**	*passed*	**15.00**	*passed*	**15.00**	*passed*

^a^ dev.—deviation; ^b^ based on target dose (*M*).

## Data Availability

The original contributions presented in this study are included in the article. Further inquiries can be directed to the corresponding authors.
